# Feature extraction via similarity search: application to atom finding and denoising in electron and scanning probe microscopy imaging

**DOI:** 10.1186/s40679-018-0052-y

**Published:** 2018-03-01

**Authors:** Suhas Somnath, Christopher R. Smith, Sergei V. Kalinin, Miaofang Chi, Albina Borisevich, Nicholas Cross, Gerd Duscher, Stephen Jesse

**Affiliations:** 1 The Institute for Functional Imaging of Materials, Oak Ridge National Laboratory, Oak Ridge, TN 37801 USA; 2 The Center for Nanophase Materials Sciences, Oak Ridge National Laboratory, Oak Ridge, TN 37801 USA; 3 Materials Science and Technology Division, Oak Ridge National Laboratory, Oak Ridge, TN 37801 USA; 4 Department of Materials Science and Engineering, University of Tennessee, Knoxville, TN 37996 USA

**Keywords:** Electron microscopy, Image denoising, Atom finding, Scanning transmission electron microscopy, Scanning tunneling microscopy, Singular value decomposition, Clustering, Statistics

## Abstract

We develop an algorithm for feature extraction based on structural similarity and demonstrate its application for atom and pattern finding in high-resolution electron and scanning probe microscopy images. The use of the combined local identifiers formed from an image subset and appended Fourier, or other transform, allows tuning selectivity to specific patterns based on the nature of the recognition task. The proposed algorithm is implemented in Pycroscopy, a community-driven scientific data analysis package, and is accessible through an interactive Jupyter notebook available on GitHub.

**Electronic supplementary material:**

The online version of this article (10.1186/s40679-018-0052-y) contains supplementary material, which is available to authorized users.

## Background

Recent advances in (scanning) transmission electron microscopy (STEM) and scanning probe microscopy (SPM) made atomically resolved imaging of solids and surfaces routine [1–5]. STEM enables the visualization of the atomic structure in a broad range of materials from oxides to semiconductors and metals, and in many cases allows observation of the evolution of structure under reactive conditions or thermal stimulations [6–11]. Similarly, ultra-high vacuum (UHV) and liquid SPM modes were used to resolve atomic structures of metal and semiconductors, ad atom structures, etc. [12–16].

Both for (S)TEM and SPM, of interest is the fundamental analysis of materials physics and chemistry from imaging data. Indeed, until recently atomically resolved images were used solely to establish the local structure of materials and make qualitative observations on its quality, the presence of specific defects, etc. The progress in spatial resolution and related information limit enabled quantitative description of images, where (for STEM) atomic coordinates for some (or all) constitutive atoms can be extracted with picometer precision. Once available, this information can be used to reconstruct physical order parameter fields such as polarization [17–20], octahedral tilts [21–23], or chemical expansion [24]. Alternatively, local atomic configurations can be analyzed in an unbiased manner via statistical methods, providing information on local crystallography [25, 26]. Moreover, crystallographic information can be extracted from the shape of the atomic column [22, 27]. Parenthetically, the extraction of physical information from atomically resolved imaging data, along with 3D imaging, provides the primary stimulus for development of progressive high-resolution STEM platforms [1]. Similar approaches can be applied to scanning probe microscopy data [25, 28], albeit in this case the origin of the contrast is more complex.

These applications necessitate the development of robust and reliable techniques to extract atomic coordinates from atomically resolved images, requiring little or no human supervision. These generally require a combination of feature extraction methods with physics-based deconvolution. For STEM data, especially annular dark or bright field images, the analysis can be significantly simplified under the assumptions that the regions with maximum contrast correspond to atomic coordinates. For more complex cases, the target is the development of feature classification and feature extraction schemes. Here, we develop an approach based on sliding window decomposition and similarity search that enables fast and robust analysis of images with multiple periodic textures and can be used for denoising, feature extraction, and segmentation of data. We further note that in certain cases, the proposed algorithm leads to physically significant decompositions—however, we defer these studies to follow-on work focused on specific studies of imaging phenomena.

## Methodology

**Fig. 1:**
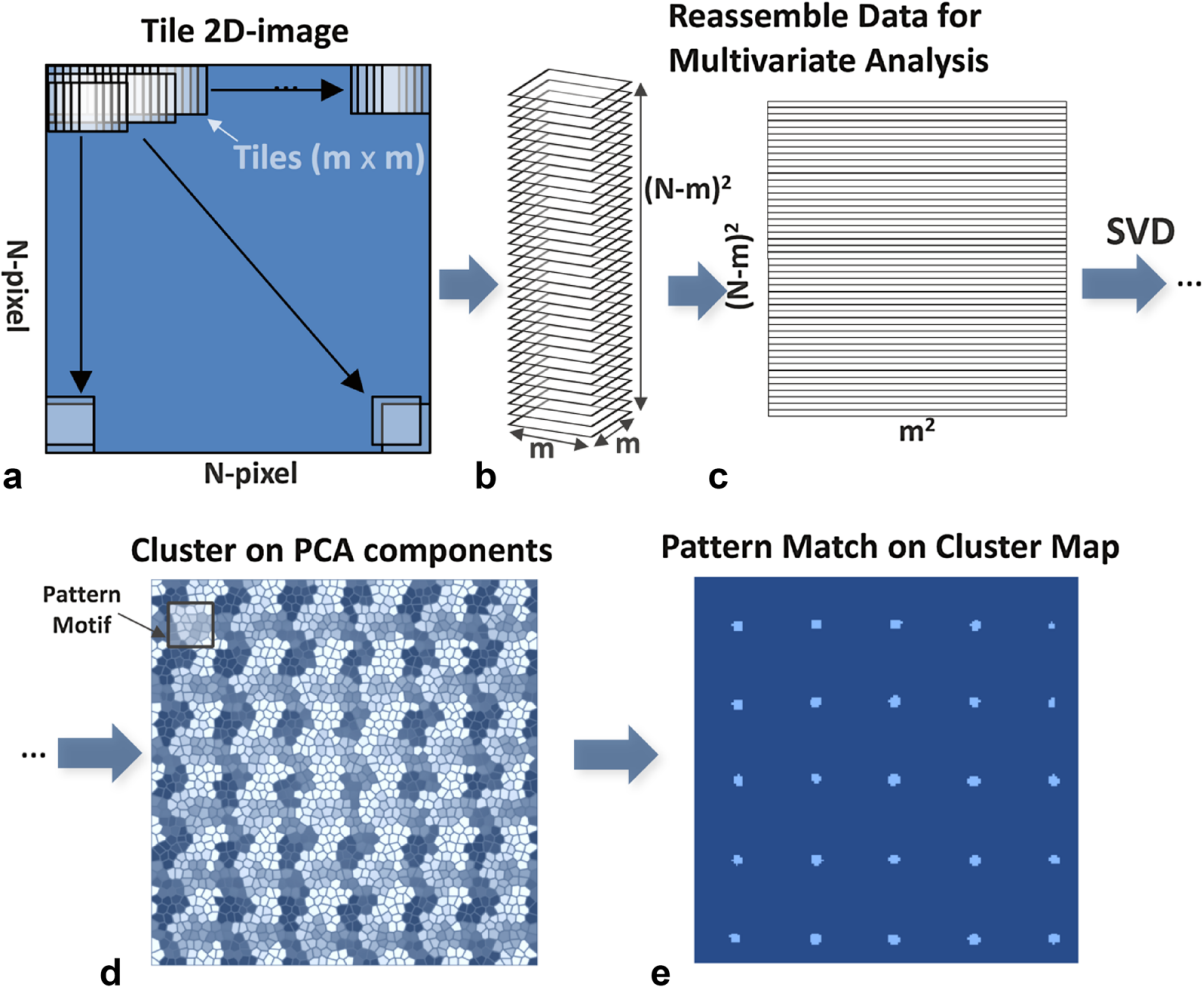
Schematic illustrating the fundamentals of the singular value decomposition (SVD)-based image denoising technique and the pattern matching-based techniques for identifying atoms in images. **a** The denoising process starts with sliding a small window across the given image column-by-column and then row-by-row. **b** A stack of (*N* − *m*)^2^ windows, each with *m* × *m* pixels, is built by copying the contents of the window at each location. **c** This 3D stack of windows is flattened to a 2D matrix by flattening the *m* × *m* pixel windows to 1D arrays with *m*^2^ elements. SVD is performed on this 2D matrix to decompose the data into the most correlated and least uncorrelated (noise) components. The image is denoised by reconstructing the 2D matrix in **c** with only the most correlated SVD components and reversing the steps from **c** to **a**. **d** *K*-means clustering on the SVD results groups pixels exhibiting similar trends together in a cluster label map. Representative examples of repeating patterns or motifs in the label map are selected for pattern matching. **e** Each motif is compared to every section in the label map to generate a pattern matching scores’ map. Each continuous-valued scores map is thresholded to generate binary maps with segments and the centroids of these segments provide the coordinates of the repeating patterns such as atoms

Figure 1a–c shows the fundamentals of the proposed approach, including denoising, feature-based clustering, and similarity search. The proposed algorithm leverages the fact that noise within different sections of the image is typically uncorrelated. Thus, images can be denoised by only retaining strongly correlated information and removing poorly correlated or uncorrelated information. To compare each section of an *N* × *N* pixel image with every other section of the image, we use an *m* × *m* pixel frame or window where *N* is much larger than *m*. For atomically resolved images, an optimal value for *m* can be calculated using information about the periodicity of the lattice [29]. See Figures S1 and S2 in Additional file 1 for examples of the original *N* × *N* pixel image and a corresponding *m* × *m* pixel window. The generated *m* × *m* pixel frame is slid across the original image column-by-image column and then image row-by-image row. At each position, the *m* × *m* pixels within the frame are copied to construct a stack of (*N* − *m*)^2^ windows each with *m* × *m* pixels.

As a second step, each window in the stack is flattened from an *m* × *m* two-dimensional matrix to a one-dimensional array containing *m*^2^ elements. When it is desired to emphasize subtle changes in the atomic periodicity in certain atomically resolved images, additional information, such as the magnitude of the fast Fourier transform of the window, can be appended to the window. This combination of real-space window and corresponding FFT with certain weighting factor allows one to balance relative weight of real- and reciprocal space features and greatly improves flexibility of the analysis. We further note that for different preponderant textures, other image transforms can be used, e.g., Radon transform for analysis of the linear domain structures. Overall, this operation transforms the three-dimensional stack of windows into a large two-dimensional matrix [(*N* − *m*)^2^ windows, each with *m*^2^ pixels].

Subsequently, we apply singular value decomposition (SVD) to identify the most and least correlated trends in the windows. Briefly, SVD breaks up a dataset into orthogonal components arranged in decreasing order of statistical significance as

1 $$A_{pq} = U_{pr} S_{r} V_{qr}^{\text{T}} ,$$

where *A* is the dataset of interest containing *p* windows with *q* pixels, *V* describes the *r* most significant components, each containing *q* pixels. *U* describes the abundance of these *r* components in the *p* windows and *S* the variance or statistical importance of each of the *r* components.

Consequently, the first few SVD components contain the most important correlations while the last few components contain the least correlated information and are typically considered to be noise. Figures S3–S5 in Additional file 1 show results from SVD applied to a windowed dataset. The original dataset, *A*, can be reconstructed exactly from the above equation or partially by using a subset of the *r* components. Thus, the 2D (*N* − *m*)^2^ × *m*^2^ dataset can be reconstructed using only the most informationally significant components from SVD. Subsequently, the windowing process shown in Fig. 1a–c can be used in reverse to generate the filtered image. Figure S6 in Additional file 1 shows the reconstruction of an image using the first few SVD components. Though the windowing process is shown for a square *N* × *N* pixel image, the same procedure can be used on rectangular images as well.

The results from SVD are further used to identify atoms or atomic columns via a pattern matching approach outlined in Fig. 1d–e. We begin the atom finding process by performing *k*-means clustering on *U* or a subset of *U*. *k*-Means clustering classifies data points into *k* clusters by Euclidean distance such that the variance within each cluster is minimized. In other words, *k*-means groups data points or pixels such that pixels within the same cluster are more similar to each other than those in other clusters. Applying *k*-means to *U* results in a (spatial) map of labels where the value at each pixel is the index of the cluster that the pixel belongs to *k*-means can also be applied to a subset of *U* to discount SVD components whose eigenvectors do not exhibit regular patterns. These components often contain information regarding long-range features (e.g., − drift), instrument noise (e.g., − 60 Hz noise), etc. Such manual selection of components in *U* can better enable *k*-means to capture the desired features. *k*-means requires *k* to be specified a priori and it is a challenge to determine an appropriate value of *k* that best represents the data. Hence, we ‘over-cluster’ the dataset, or choose a large value for *k* (e.g., – 24 to 60) to allow *k*-means to capture the finer nuances in the image, such as phase boundaries, in the original image.

Next, we manually select *t* square or rectangular windows in the denoised image that are centered on repeating patterns, such as atoms or atomic columns. Separate motifs are selected to represent each of the families of atoms or atomic columns. The coordinates of these windows are used to extract a corresponding set of *t* motifs from the spatial map of cluster labels obtained from *k*-means. The spatial abundance of each motif is calculated by scanning the motif across the spatial map of cluster labels, image column by image column and then image row by image row. For a given motif at a given location on the cluster label map, the ‘matching score’ is calculated as the number of pixels in the motif that match with the current window in the cluster label map. This matching score is divided by the number of pixels in the motif such that the score always ranges from 0 to 1. In the event that two motifs identify the same set of atoms, one of the motifs is removed. Additional motifs may be required to identify those atoms that are not captured by the original set of motifs. One example is the case where drift in the microscope results in distortions in the shapes of atoms in certain sections of the image.

This matching process results in *t* spatial maps of matching scores corresponding to each of the *t* motifs. These continuous-valued spatial maps of matching scores are manually thresholded to generate *t* binary maps where the score is set to 1 if the matching score is greater than the threshold and 0 otherwise. The threshold values for each pattern are manually chosen such that the number of matched areas is maximized while minimizing any overlap with segments from other patterns. For atomically resolved images, the coordinates of atoms can be estimated by calculating the centroid of each segment from the thresholded maps. When the same atom is identified for multiple motifs, supervised machine learning techniques such as *K*-nearest neighbors are used to remove duplicates and assign the atom to the correct motif. Subsequently, a variety of approaches can be used to refine the positions of the atoms for further analysis as necessary [23, 25, 28].

## Results

**Fig. 2:**
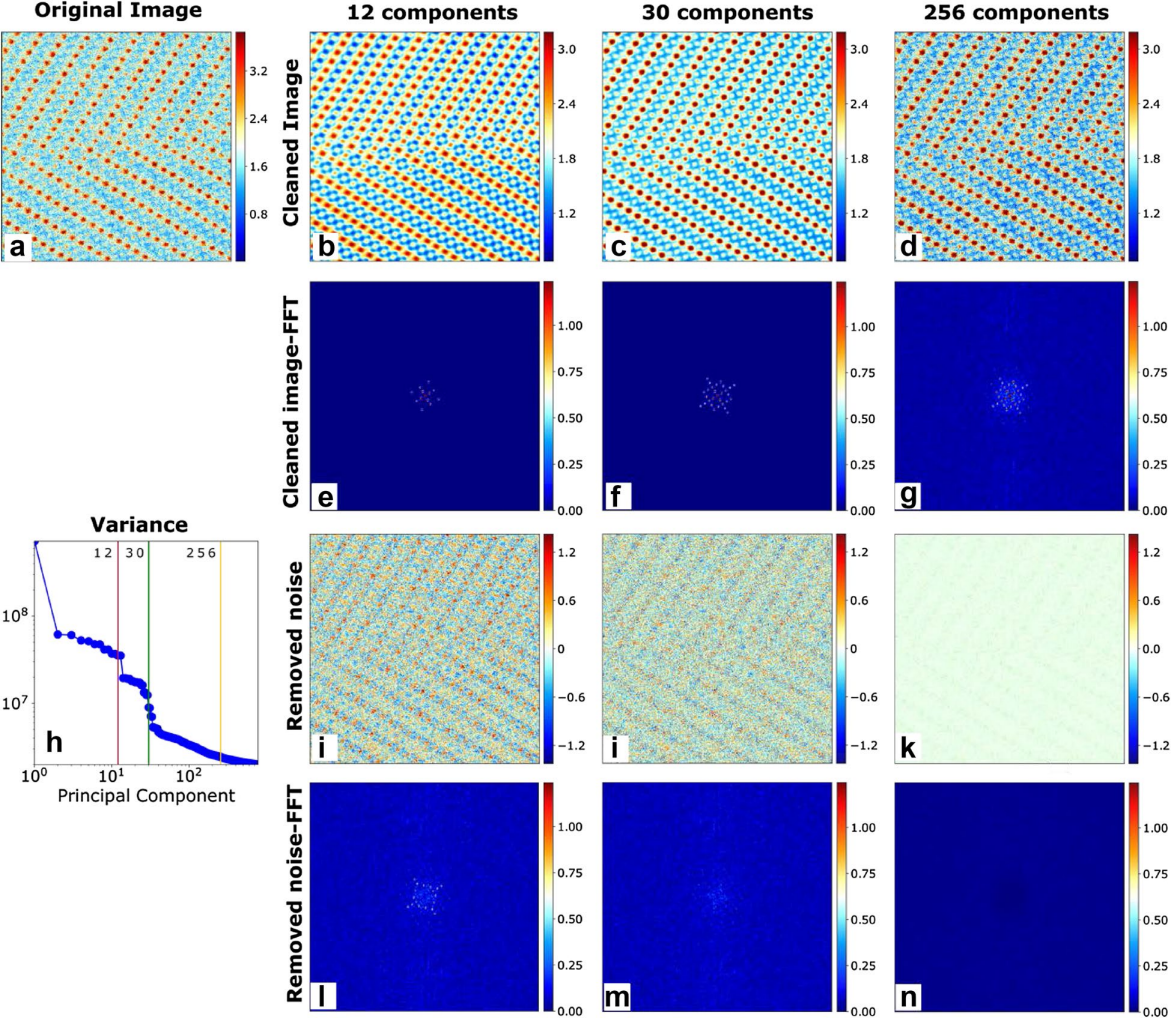
Choosing the appropriate number of SVD components to effectively denoise images. **a** Original atomically resolved image. Denoised images obtained by reconstruction using **b** 12, **c** 30, and **d** 256 SVD components. Magnitudes of the 2D FFTs of images reconstructed using **e** 12, **f** 30, and **g** 256 SVD components. **h** Variance of the SVD components showing the cut-offs for (red) 12, (green) 30, and (cyan) 256 SVD components as vertical lines. Removed noise, calculated as the difference between the original and denoised images, when reconstructing images using **i** 12, **j** 30, and **k** 256 SVD components. Magnitudes of the 2D FFTs of the removed noise when reconstructing images using **l** 12, **m** 30, and **n** 256 SVD components

The selection of the appropriate number of components to reconstruct the image plays a very important role in the denoising process. Figure 2 shows the denoising process applied to an atomically resolved image obtained from a scanning transmission electron microscope (STEM). The image shows a clear lattice structure with some bright and some dim spots signifying atomic columns. As is the case in most annular dark-field STEM images, certain columns of atoms result in a signal that is often comparable to or below the noise floor, which makes it very challenging to identify these atomic columns. After applying the windowing algorithm, we applied SVD to the windowed dataset and reconstructed images with varying number of SVD components. Figure 2h shows that variance, or statistical significance, of components decreases exponentially with the number of components. In fact, the first 32 of the 180,625 components contain nearly all the statistically and physically relevant information. Reconstructing the dataset with only the first eight components resulted in an image with minimal noise. Though the dominant lattice structure is certainly visible in the cleaned image in Fig. 2i, l shows that finer shifts in the atomic positions are lost. The fast Fourier transform (FFT) of these images shows that the cleaned image only captures the signal from a few lower order peaks while information from several higher order peaks are lost. When reconstructing the dataset with the first 700 components, we see that the cleaned image is comparable to the original image since only a few components were removed during the reconstruction. Consequently, Fig. 2k shows that minimal information was discarded and the vast majority of information in the frequency space is retained in Fig. 2g. Reconstructing the dataset with only the first 32 components resulted in an image that retains the general lattice structure and the finer shifts in the atomic columns while discarding only the noise as seen in Fig. 2c, j. We reiterate that the image can be reconstructed using any subset of components (for example—components 2,5,8,9,15….) and not only using the first few components. Thus, it is critical that the appropriate components be chosen to reconstruct the images.

**Fig. 3:**
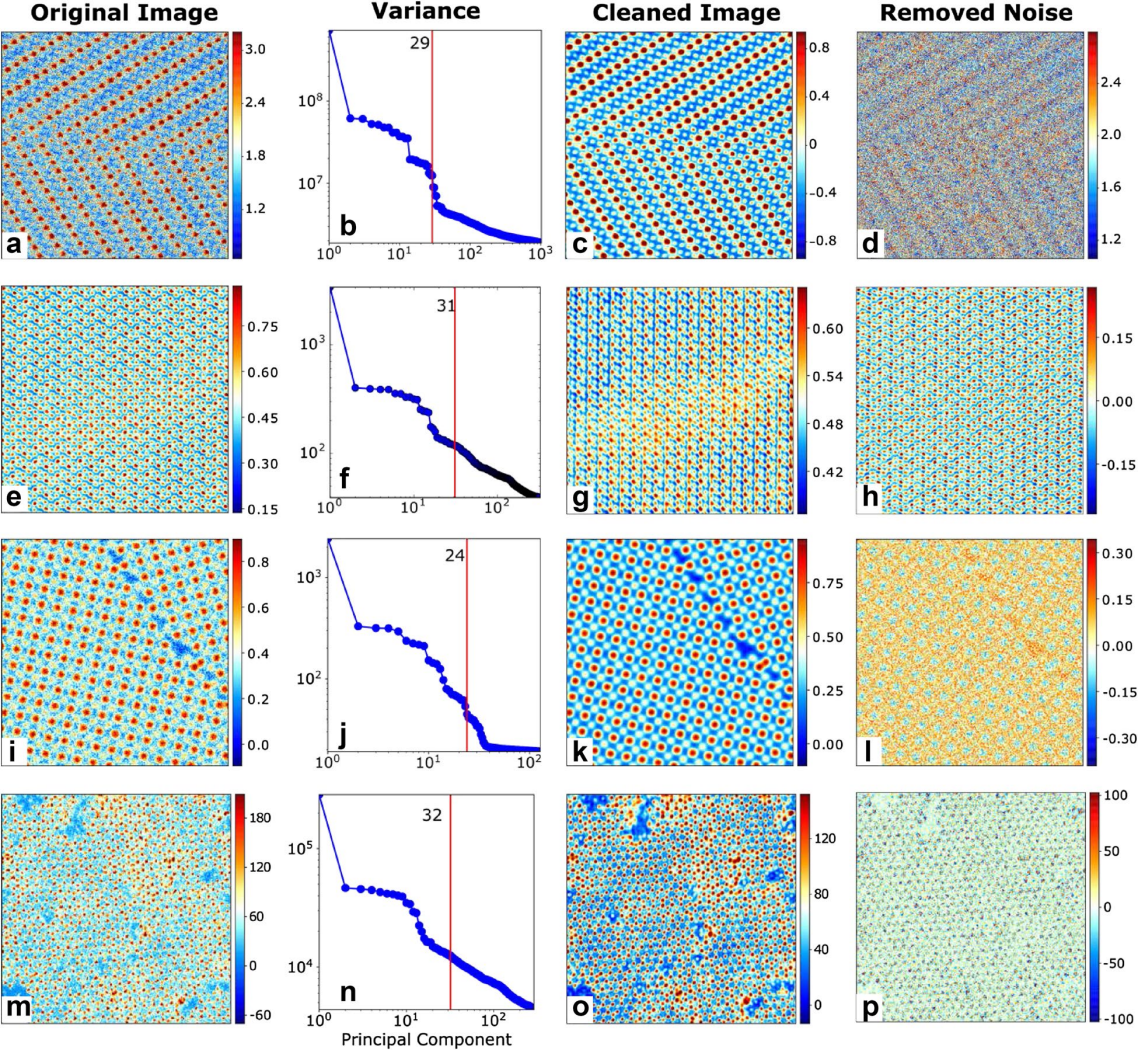
Image windowing and SVD-based denoising algorithm applied to four atomically resolved images. **a**, **e**, **i**, **m** Original images showing varying levels of noise, number of atoms, and presence of contaminants. **b**, **f**, **j**, **n** Variance of the SVD components and the number of components used to reconstruct images shown by the red line. **c**, **g**, **k**, **o** Denoised images obtained by reconstructing using a subset of the SVD components. **d**, **h**, **l**, **p** Noise removed from images, calculated as the difference between the original and denoised images

Figure 3 shows this windowing and SVD-based filtering methodology applied to four atomically resolved images obtained from STEM and scanning tunneling microscopes (STMs). The sample description is provided in “Methods” section. In all cases, the original images show significant amount of noise that makes it challenging to identify certain atoms or columns. SVD on the windowed datasets resulted in components whose variance decreased exponentially with increasing number of components as seen in the variance plots. The denoised images were generated by reconstructing the windowed datasets using only the first 24–32 components from the complete 500–2000 components. In all cases, the denoised image clearly reveals the atomic columns regardless of the intensity of the signal. The removed signal mainly contains the uncorrelated noise in the original image. The denoised images are neither missing atoms nor do they have additional atoms that were not present in the original image. As this figure shows, our denoising approach works for images with different numbers of atoms, images with defects, images with shifts in the atomic periodicity caused by phase boundaries or grain boundaries (Fig. 3a, e, i), and images of surfaces with contaminants (Fig. 3m).

**Fig. 4:**
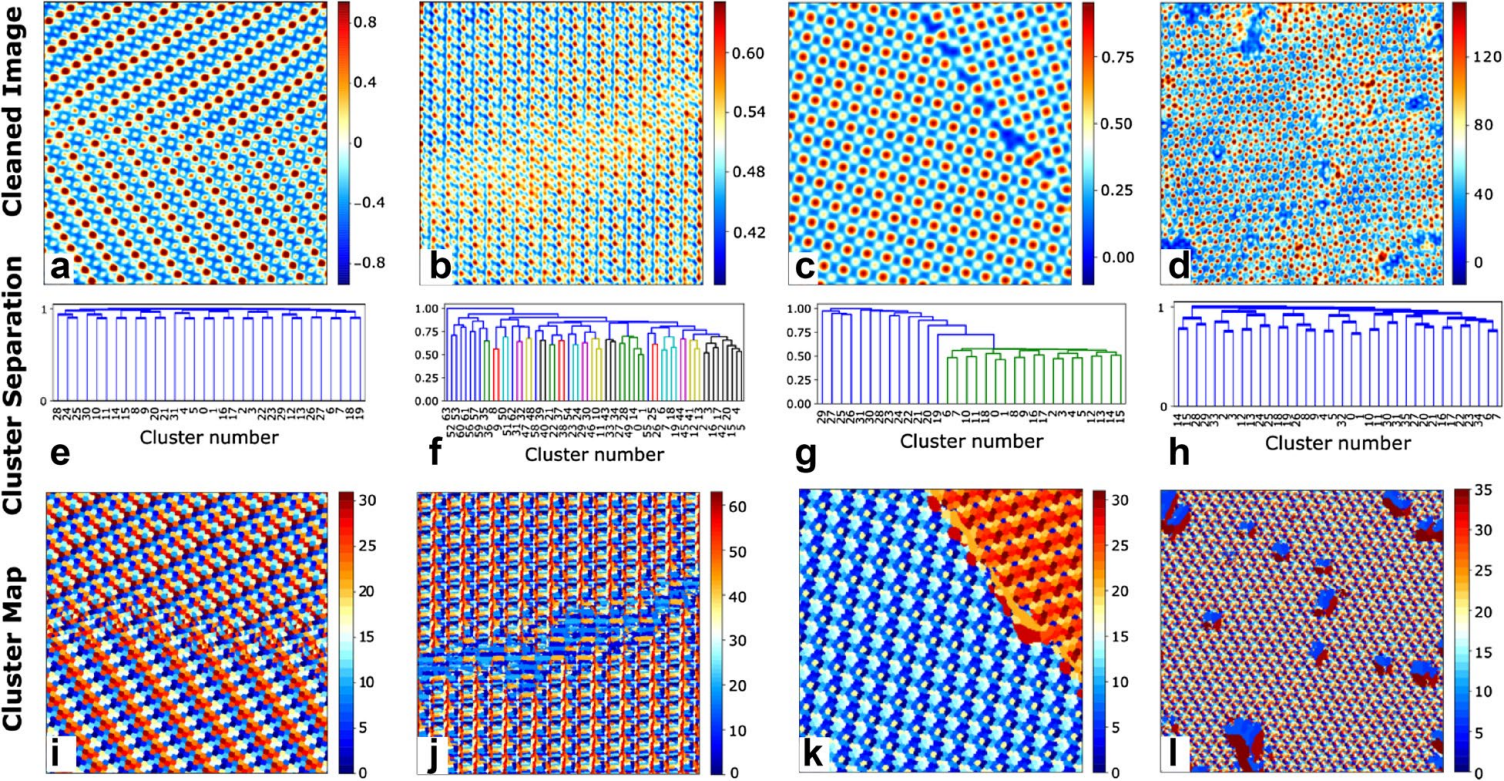
*k*-Means clustering applied to SVD results to identify trends in the dataset that can be invisible to the human eye. *K*-means clustering is applied to a subset of the *U* matrix obtained from SVD on the windowed dataset. **a**–**d** Images denoised using the windowing and SVD-based denoising algorithm. **e**–**h** Dendrograms showing the grouping of different clusters. The distance between two clusters is given by the height of the horizontal line connecting two clusters. **i**–**l** Maps of cluster labels obtained from *K*-means clustering. These maps show phase boundaries, cracks in the crystal structure, grain boundaries, and locations of contaminants in the image

Following the denoising, the results from SVD on the windowed dataset can also be used to identify atoms via the pattern matching approach described above. The first and most challenging step in this process is configuring *k*-means clustering such that it effectively identifies subtle trends such as phase boundaries, domain walls, grain boundaries, dislocations, or cracks and segregates the dataset accordingly. Figure 4 shows results of *k*-means clustering applied to the *U* dataset obtained from SVD on the windowed dataset. Components containing long-range features and changes in background intensities were discarded in most cases for effective identification of subtle features. For the images in Fig. 4a, b, the absolute value of the FFT of the windows was appended to the windows to emphasize subtle changes in the periodicity in the image. These careful considerations enabled *k*-means to differentiate phase regions in the left and right side of Fig. 4a that are invisible to the human eye. Correspondingly, the cluster label map in Fig. 4i shows different patterns on the left and right separated by a red section in the center. Similarly, *k*-means was able to clearly identify the antiphase boundary in Fig. 4b as seen by the broad blue “crack” dividing the top and bottom of Fig. 4j. *k*-Means also clearly identified the grain boundary in Fig. 4c and the defects/impurities in Fig. 4d.

**Fig. 5:**
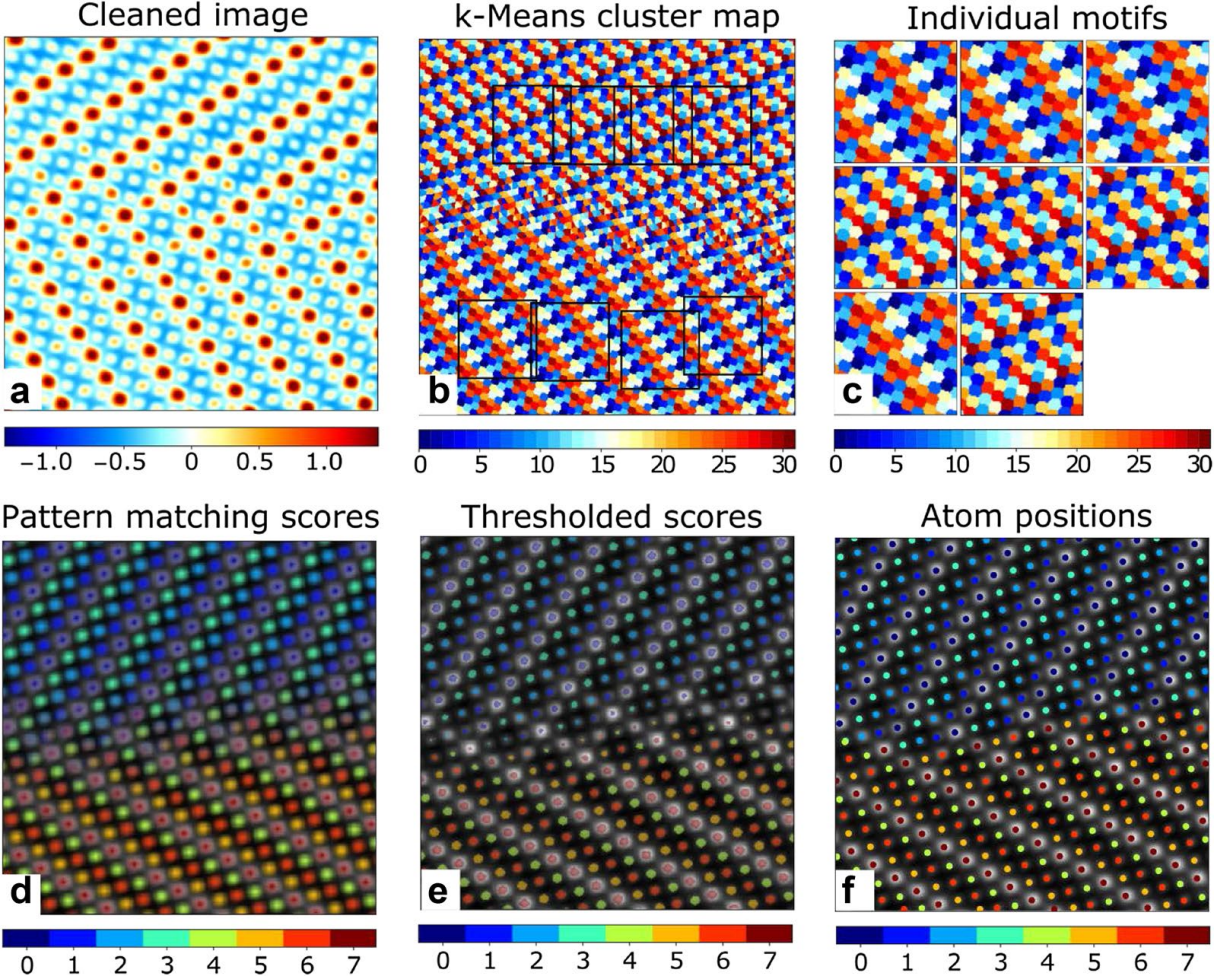
Pattern matching approach for identifying and classifying atoms. **a** Image denoised using the denoising algorithm presented in this paper. **b** Map of cluster labels obtained from *k*-means clustering on a subset of the components obtained via SVD on the windowed dataset. Patterns to be used for pattern matching are shown by the black squares in **b** and are expanded in **c**. **d** Overlays of pattern matching scores ranging from 0 to 1 for each pattern. Results for the different motifs are distinguished by color. The color is set to transparent for a matching score of 0 and to the solid color corresponding to the motif for a matching score of 1. **e** Binary maps of pattern matching scores after thresholding to either 0 or 1. **f** Positions of the atoms identified for each motif obtained from the centroids of the segments in **e**. The **d** pattern matching scores, **e** thresholded scores, and **f** atom positions have been superimposed over a black-and-white map of the denoised image shown in **a**

Following successful clustering, this pattern matching technique can be applied to identify and differentiate atoms as shown in Fig. 5. The denoised image in Fig. 5a was reconstructed by carefully choosing a subset of the first 28 SVD components. *k*-Means clustering was performed on the same set of components to provide the map of cluster labels shown in Fig. 5b. Four motifs, centered on the two different classes of atomic columns, were manually chosen from the cluster label map, demarcated by the black squares in Fig. 5b and also shown in Fig. 5c. Figure 5d shows the overlay of the normalized matching scores for each of the four motifs and the atoms identified by different motifs are differentiated by color. We observed minimal overlap or mis-identification of the atoms at this stage and only 4 of the 300+ atoms were not identified due to artifacts in the center of the *k*-means cluster map. Independently thresholding the pattern matching scores maps resulted in binary maps shown in Fig. 5e. The coordinates of the atoms were calculated via the centroid of each segment in Fig. 5e.

## Discussion

The microscopy community has developed and used a variety of techniques for denoising images and finding the positions of atoms and atomic columns in images since the inception of the SPMs and STEMs [30]. Among the many techniques for denoising atomically resolved images, Gaussian blurring, filtering in the Fourier space, averaging over multiple unit cells, and averaging over a stack of images are some of the most commonly used methods. Most of these techniques are fast and simple but have major shortcomings. For example, filtering in the Fourier space is prone to adding additional atoms at vacancies, removing displacements in atomic positions, etc. Furthermore, it is challenging to further fine-tune the filter since the frequencies with significant information vary from image to image and are not known a priori. Cross-correlation and phase-correlation methods typically require a stack of multiple images and cannot work on a single image like our technique. The current state-of-art technique for image denoising is a non-local means (NLM) [31] technique called block-matching and 3D filtering (BM3D) [32], which identifies windows or patches that are similar, performs 3D wavelet denoising on similar patches and finally applies a Wiener filter. BM3D also shares the same shortcomings as frequency space filtering. Additional file 1: Figure S7–S10 shows the results from popular image filtering techniques. Additional file 1: Figure S11 compares the best results from five image denoising techniques. We observe that our technique is substantially better than all other techniques, including BM3D, at effectively removing the majority of the noise while retaining all the important information regarding the lattice structure. All other techniques only remove the short-range features or high-frequency components while the noise at low-frequency components still remains in the image. Furthermore, all the other techniques tend to erase atoms with relatively low intensities easily as the strength of the filter is increased.

We also compared our atom finding technique with other conventional alternatives. Similar to the image denoising alternatives, the atom finding techniques also have their own advantages and limitations. For example, though Gaussian convolution is fast and simple, it is unable to differentiate regions with different lattice structures and often deletes atoms with lower intensities along with the noise especially in images with low signal-to-noise ratio. Window-based convolutions on the other hand perform slightly better than Gaussian convolutions at identifying atoms with relatively low intensities but at the cost of a significant number of false-positives since the method is dominated by atoms or atomic columns with relatively high intensities. Moreover, similar to Gaussian convolution, window-based convolutions are also poor at distinguishing regions with different lattice structures. We find that our technique is superior to the aforementioned techniques since it has consistently been able to correctly identify all atoms and distinguish regions. See Additional file 1: Figure S12 for more information.

Clearly, each denoising and atom finding technique has its own merits and disadvantages and a single technique may not be ideal for every image. For instance, while our technique is consistent in denoising and atom identification, our (currently) computationally intensive algorithm requires modifications to make it suitable for real-time denoising of images and identification of atoms. We find that the best solution is for such algorithms to be made available freely via open-source packages to allow researchers to adopt the algorithms that suit them the best.

## Conclusions

We implemented methods for finding atoms and patterns in the high-resolution images based on similarity search on sliding transforms of images. This approach is universally applicable to STEM, STM, and AFM data, and can be also applied to the other feature- finding problems. The use of the identification object comprised image subset and appended Fourier transform allows tuning for increased detectability of periodic structures, and can be adapted to other characteristic morphologies, e.g., via use of Hough transforms.

All the image denoising and atom finding algorithms presented in this paper are freely available in our open-source, community-driven, python package—Pycroscopy (https://github.com/pycroscopy/pycroscopy). The scientific workflow presented in this paper is available via a Jupyter notebook (http://nbviewer.jupyter.org/github/pycroscopy/pycroscopy/blob/master/jupyter_notebooks/Image_Cleaning_Atom_Finding.ipynb) that allows straightforward application of the presented methodology to arbitrary images.

## Methods

The image in Fig. 2 is Li_0.33_ La_0.57_ TiO_3_ described in [30]. Image courtesy of Miaofang Chi, Oak Ridge National Laboratory.

The image in Fig. 3b is Mo-V-M oxide described in [31, 32]. Image courtesy of Albina Borisevich, Oak Ridge National Laboratory.

The image in Fig. 3i is a simulated image.

The image in Fig. 3m is WSe_2_ irradiated with He ions acquired via Nion Company UltraSTEM 100 at 100 keV. Image Courtesy of Nicholas Cross and Gerd Duscher, The University of Tennessee, Knoxville.

## Additional file

**Additional file 1.** Additional figures.
