# Supplementary material for: Feature extraction via similarity search: application to atom finding and denoising in electron and scanning probe microscopy imaging
Source: Adv Struct Chem Imaging. 2018 Mar 1;4(1):3. doi: 10.1186/s40679-018-0052-y (PMC5846807; doi:10.1186/s40679-018-0052-y)
Supplement: Supplementary file 1 — Additional file 1. Additional figures. [file 40679_2018_52_MOESM1_ESM.docx]

*Additional Information for*

**Feature extraction via similarity search: application to atom finding and denoising in electron and scanning probe microscopy imaging**

Suhas Somnath^1,2^, Christopher R. Smith^1,2^, Sergei V. Kalinin^1,2^, Miaofang Chi^2^, Albina Borisevich^1,3^, Nicholas Cross^4^, Gerd Duscher^4^, Stephen Jesse^1,2^

^1^ The Institute for Functional Imaging of Materials, ^2^ The Center for Nanophase Materials Sciences, ^3^ Materials Science and Technology Division, Oak Ridge National Laboratory, TN, USA.

^4^ Materials Science and Engineering, University of Tennessee – Knoxville, TN 37996, USA.


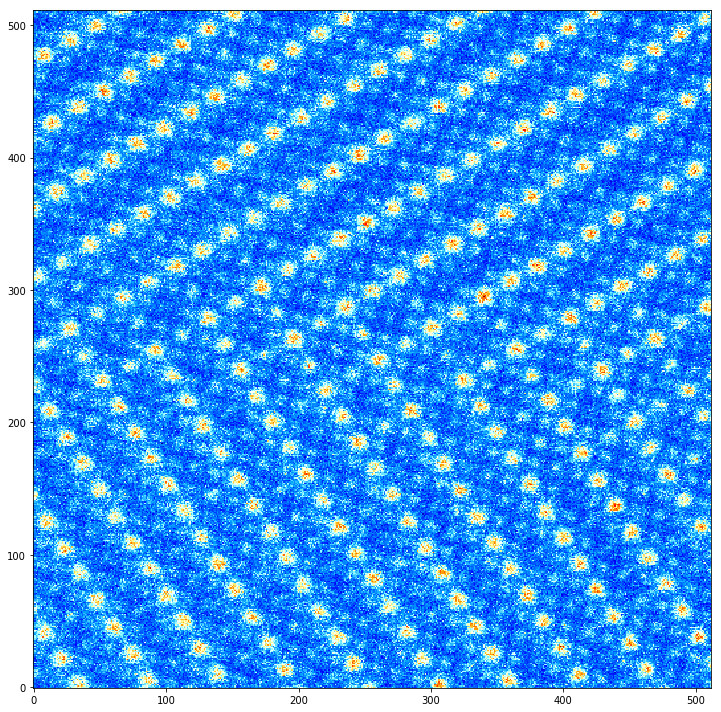


**Figure S1. Original image** - Image of Li_0.33_ La_0.57_ TiO_3_ as obtained from a scanning transmission electron microscope. The image shows significant noise which makes it very challenging to identify some of the atomic columns. The image is of size 512 x 512 pixels.


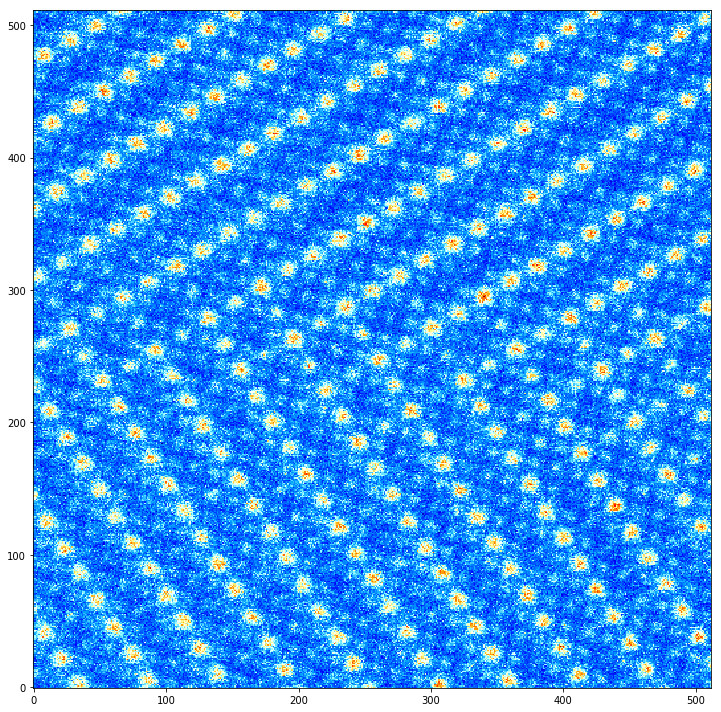


**Figure S2. Example window** - An example window extracted from the original image. The ‘windowing’ procedure, described in the main manuscript, was performed on the image presented in figure S1 to build a substantially larger windowed dataset. The image cleaning algorithm was applied to the aforementioned windowed dataset.


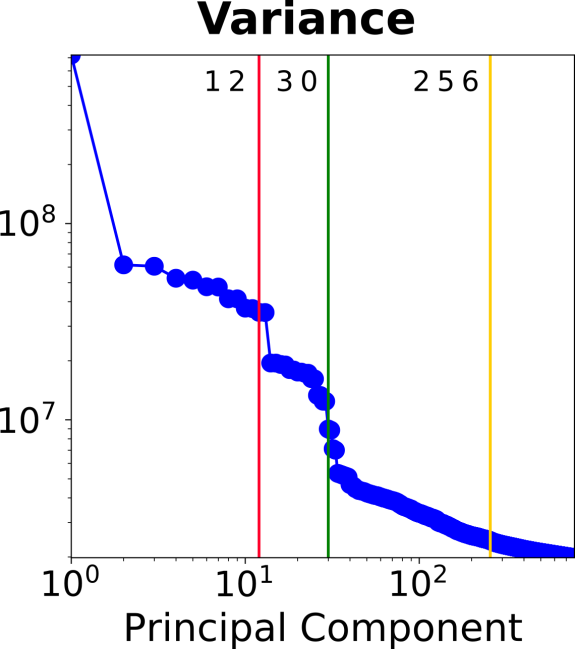


**Figure S3. SVD Variance** – Statistical significance of each of the SVD components. The components in SVD are arranged in descending order of variance or statistical significance. In other words, the first few components exhibit the most important trends in the data. The last few components show the least variance and mainly contain noise. The variance decreases exponentially with the number of components and the first 32 of the 180,625 components contain nearly all the information.


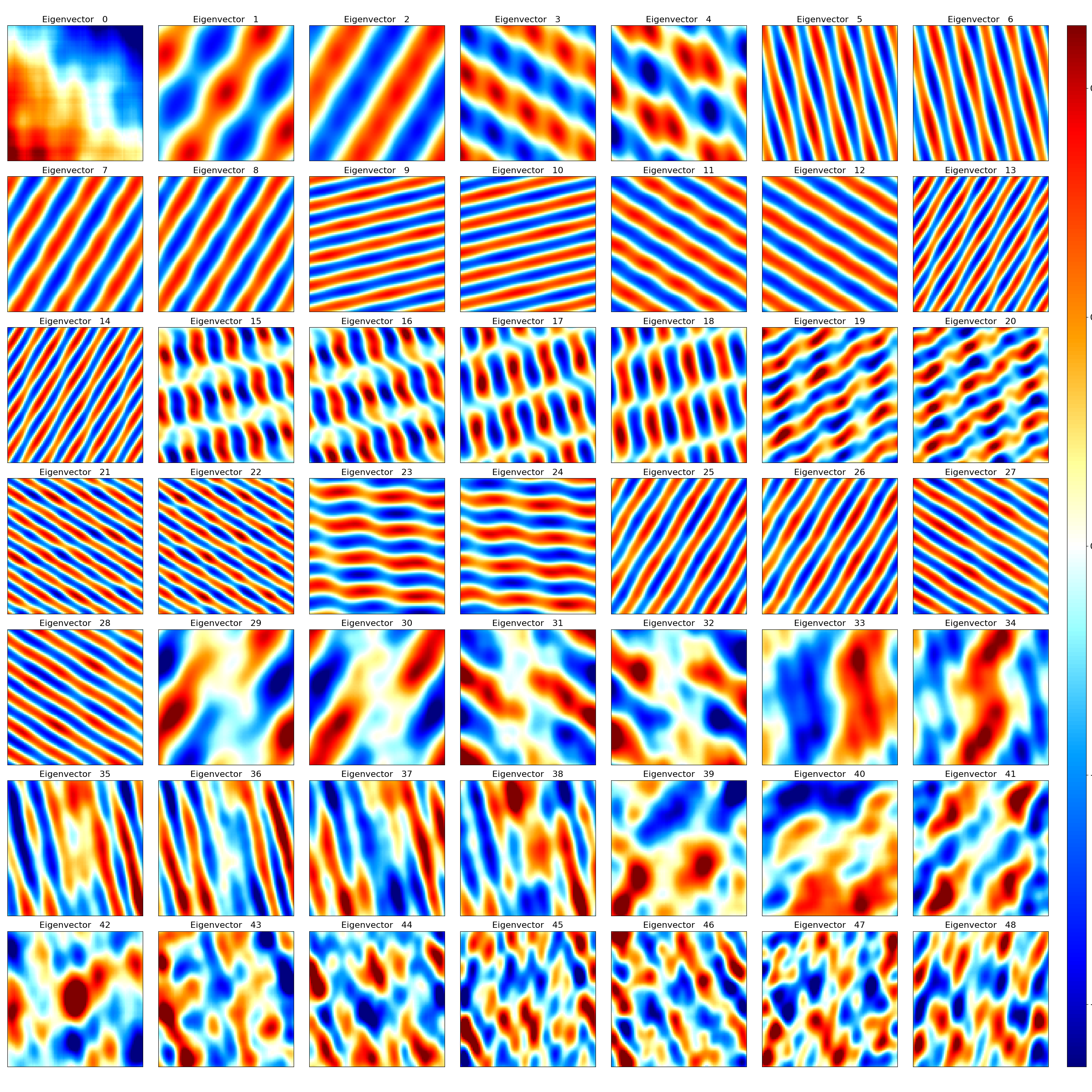


**Figure S4. SVD eigenvectors** – Eigenvectors obtained by performing SVD on the windowed dataset described in figure S2. Note that eigenvector 0 shows long-range features while all other eigenvectors show short range features. While these eigenvectors may be challenging to physically interpret, the corresponding abundance maps presented in figure S4 substantially aid the interpretation. While it may appear that senior components (components numbered 33 or greater) contain important information, figure S3 reveals that these components are likely to contain more noise than information.


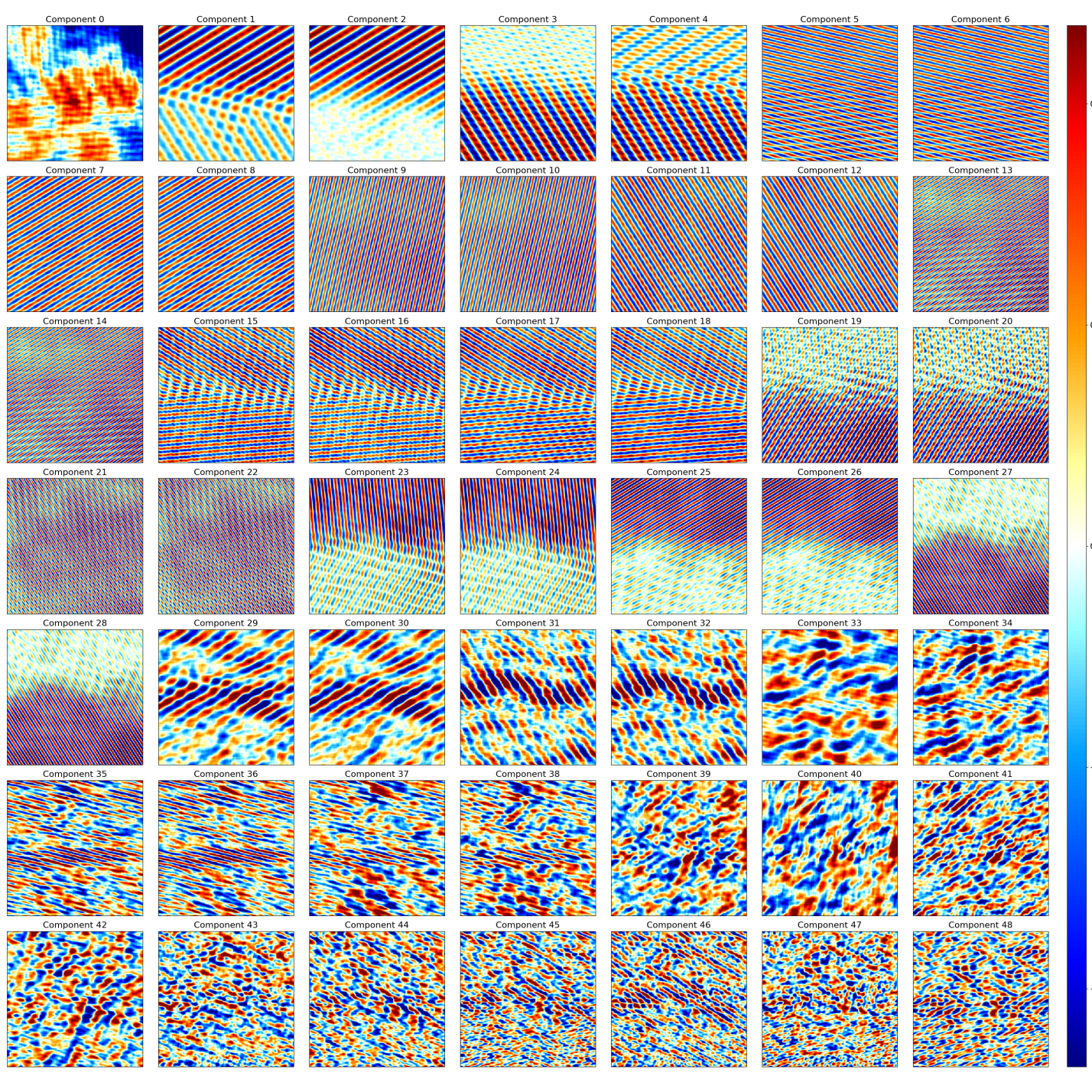


**Figure S5. SVD abundance maps** ­– Abundance maps obtained by performing SVD on the windowed dataset described in figure S2. The abundance map for each SVD component in this figure corresponds to the eigenvector for the same SVD component presented in figure S4. Note that the first component shows long range features in the image.


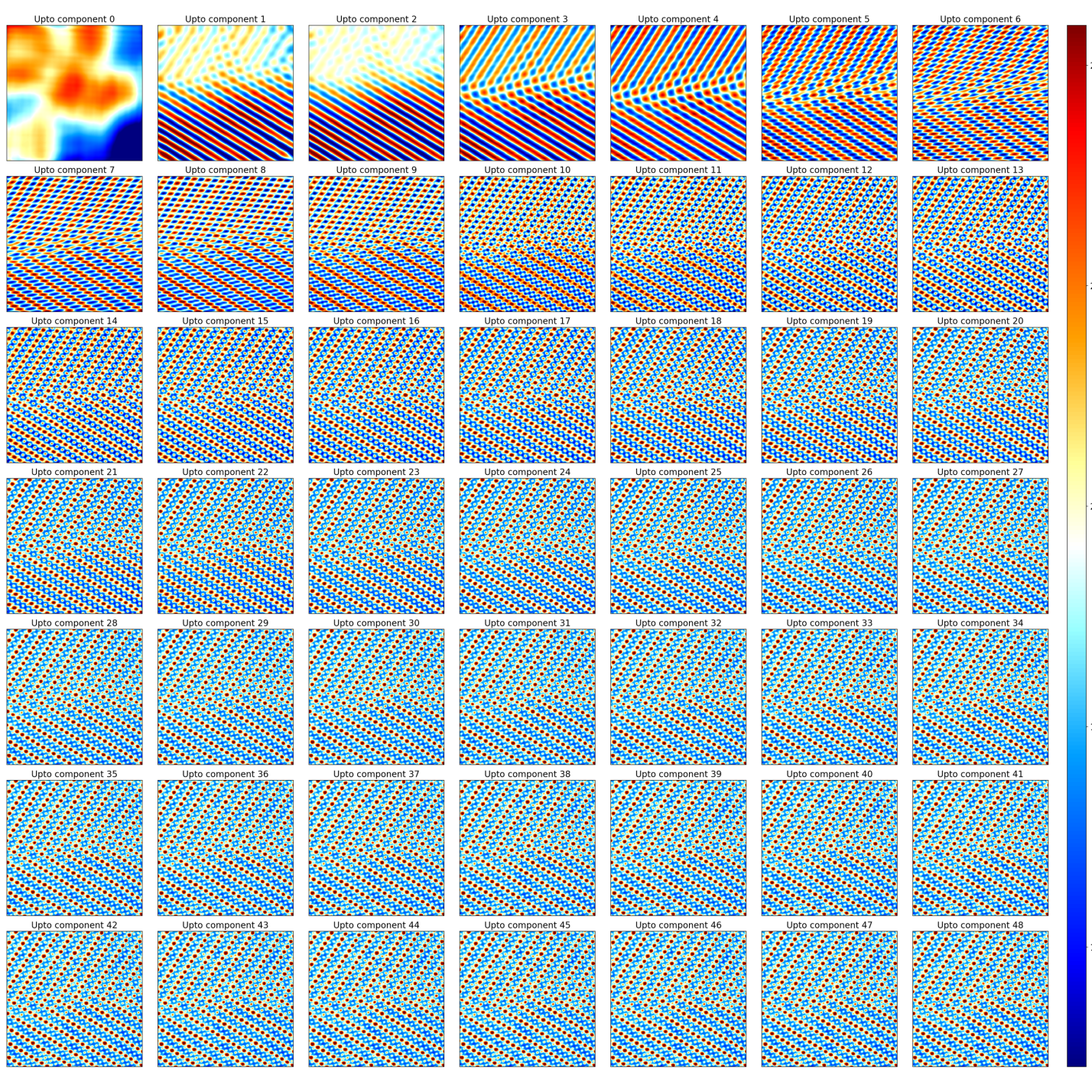


**Figure S6. Image reconstruction and denoising** – The original image can be reconstructed using a subset of the components provided by SVD. This is equivalent to setting the variance (*S* matrix) of undesirable components to zero. This figure presents the results of reconstructing the image with the first *few* SVD components. This exercise is instructive for understanding the information present in each component and for selecting the number of components to reconstruct the image with. For example, reconstructing with only the first component only captures the long-range features in the image. Reconstructing with only the first 4 components (up to component 3) captures the dominant lattice structure but finer shifts in atomic column position are lost. Reconstructing the image with the first 32 components retains the finer variations in atomic positions while eliminating most of the noise. Reconstructing with any more components only appears to add noise. Ideally, the image would be reconstructed with components 1-32 such that the long-range features are removed along with the noise.

**
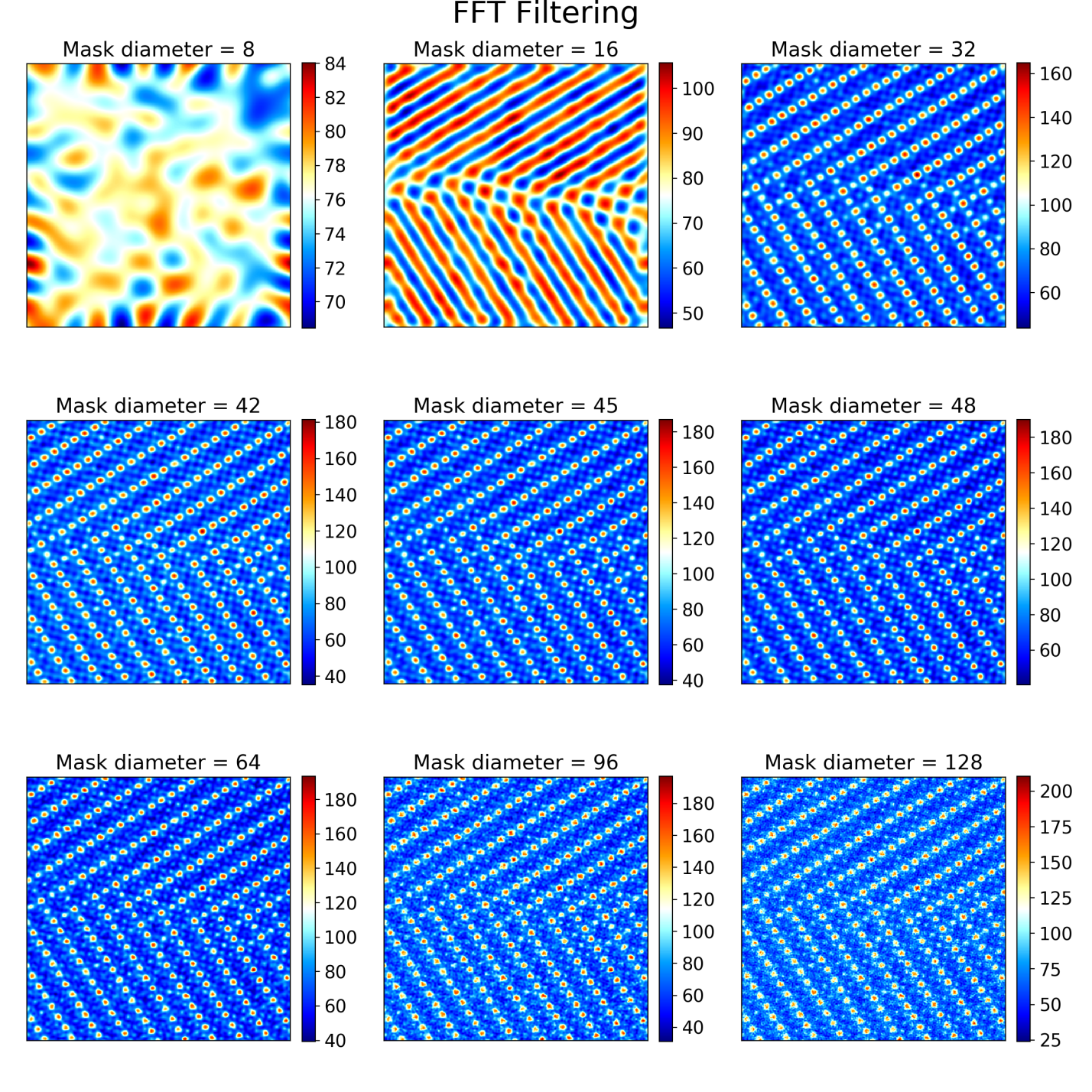
Figure S7. Image denoising via FFT Filtering** – To emulate this popular method of FFT filtering, we created a circular mask around the origin of the FFT and only retained the signal within the mask. Here we show the results of using FFT filtering using different diameters for the frequency mask applied to the image shown in figure S1. Only the long-range features are captured for mask diameters less than 20 and mask diameters greater than 45 introduce noise from higher frequencies (short range features that are smaller the inter-atom spacing). Even with the ideal mask diameter of 45, the image still contains significant noise arising from unfiltered frequencies within the mask.


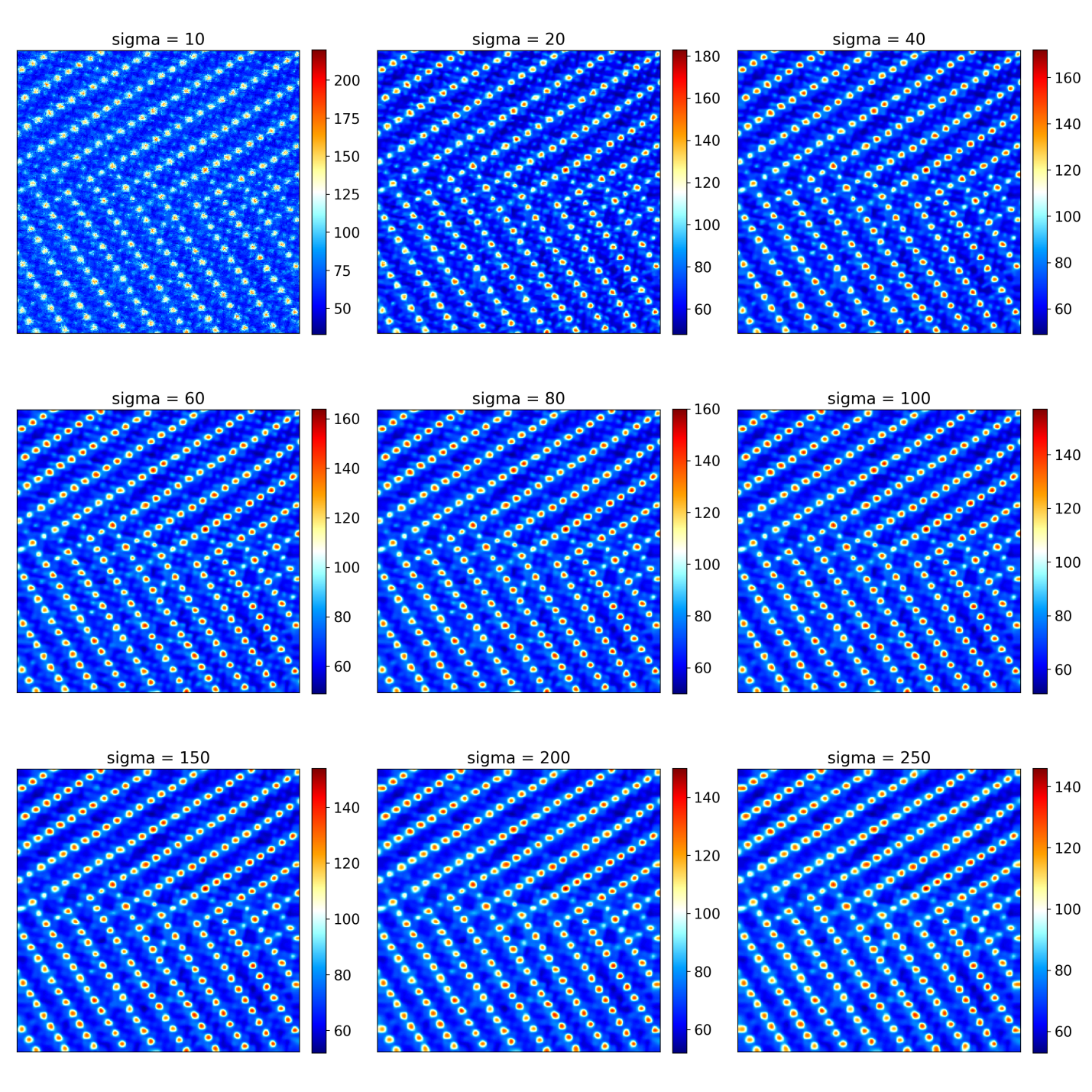


**Figure S8.** **Image denoising via the BM3D filter** – Results from the state-of-art BM3D filter applied to the unfiltered image shown in figure S1 for different sizes of the kernel (sigma). For sigma values less than 40, the filtered image still contains some amount of noise. However, for sigma values greater than 40, the atoms with relatively low intensity values begin to vanish. The filtered image obtained with sigma set to 40 still contains considerable noise from noise frequencies surrounding the dominant frequency peaks.


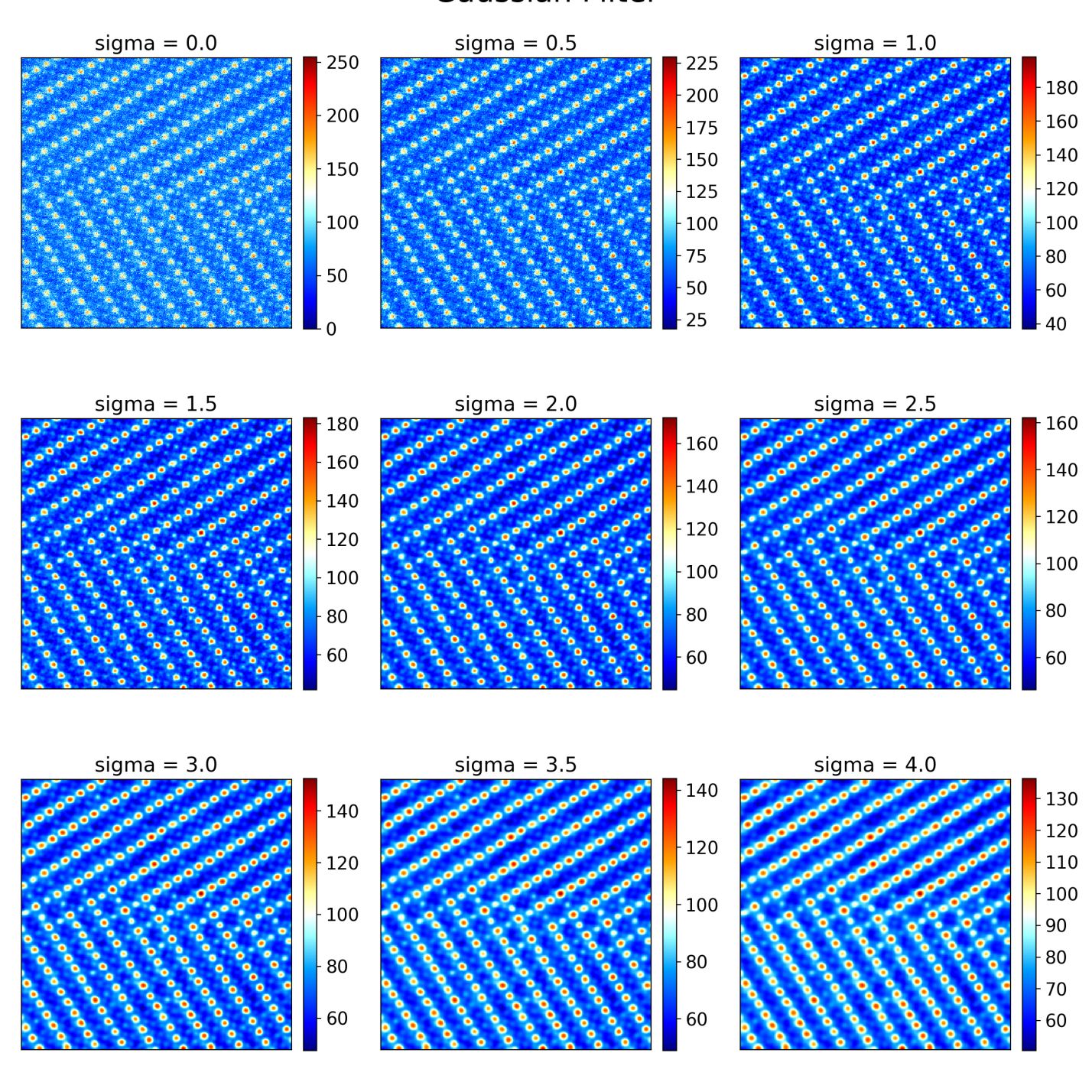


**Figure S9. Image denoising using Gaussian filters** – The conventional Gaussian filter was applied to the image shown in figure S1 where the standard deviation of the kernel (sigma) was varied over a range of values. Sigma values less than 2 were effective at removing the noise from high frequencies (very short range features). However, increasing the sigma value beyond 2.0 progressively erased atoms having relatively low intensities.


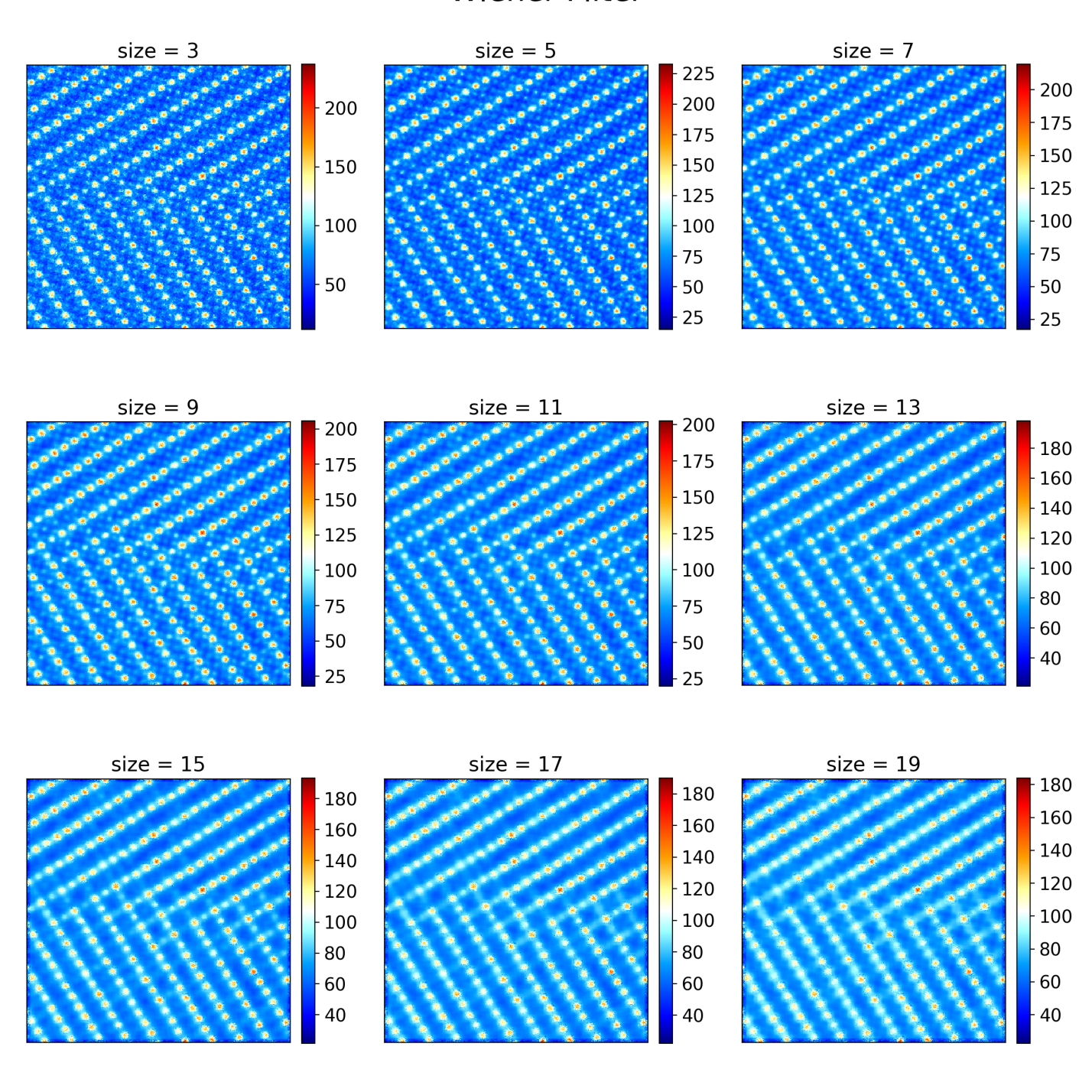


**Figure S10. Image denoising using the Wiener filter** - The Wiener filter was applied to the image shown in figure S1 where the kernel size was varied over a range of values. Kernel sizes smaller than 7 were effective at removing the noise from high frequencies (very-short-range features). However, increasing the size beyond 7 progressively erased even the relevant short-range features leaving only the long-range features in the filtered image.


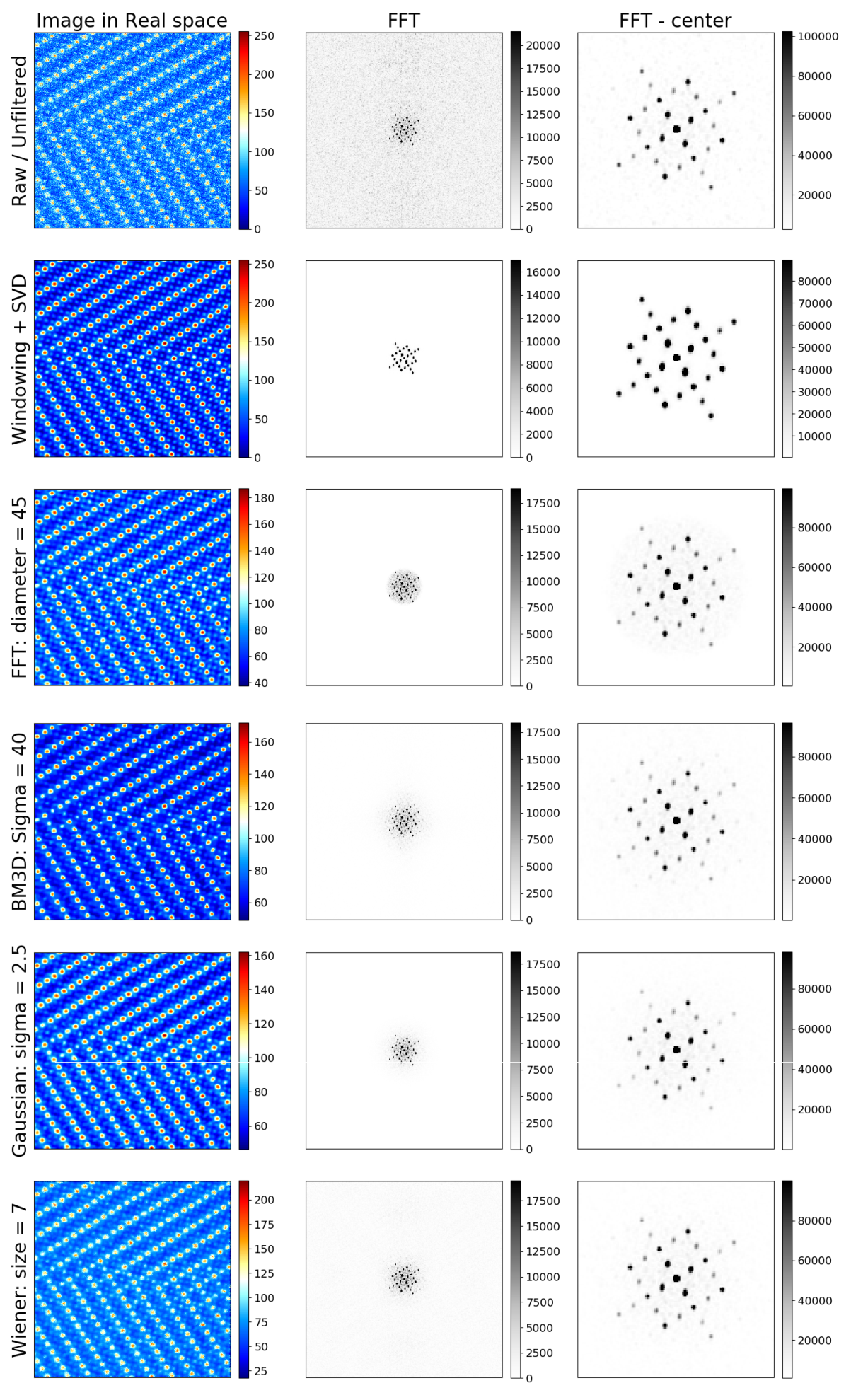


**Figure S11. Comparison of image denoising techniques** - filtered image in (left) real space and (center) frequency space. (right) expanded view of the frequency space information around the origin. Results from filtering using (top to bottom) no filter, windowing and SVD based filter described in this paper, FFT filter, BM3D filter, Gaussian filter and Wiener filter. The filters are arranged in descending order of effectiveness meaning that the windowing and SVD based filter performed best and the Wiener filter performed the poorest at denosing the image effectively without removing any important features.

| **(a)**  **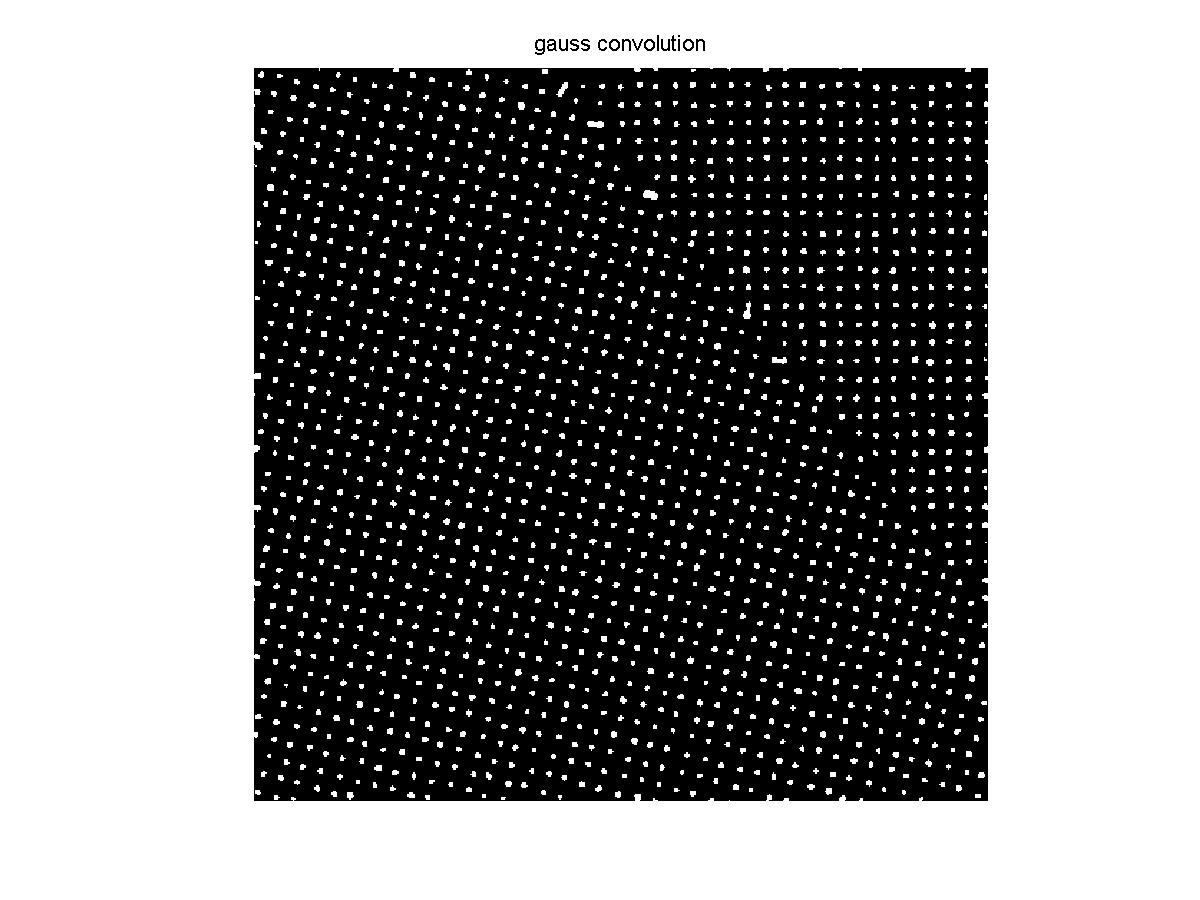** | **(b)**  **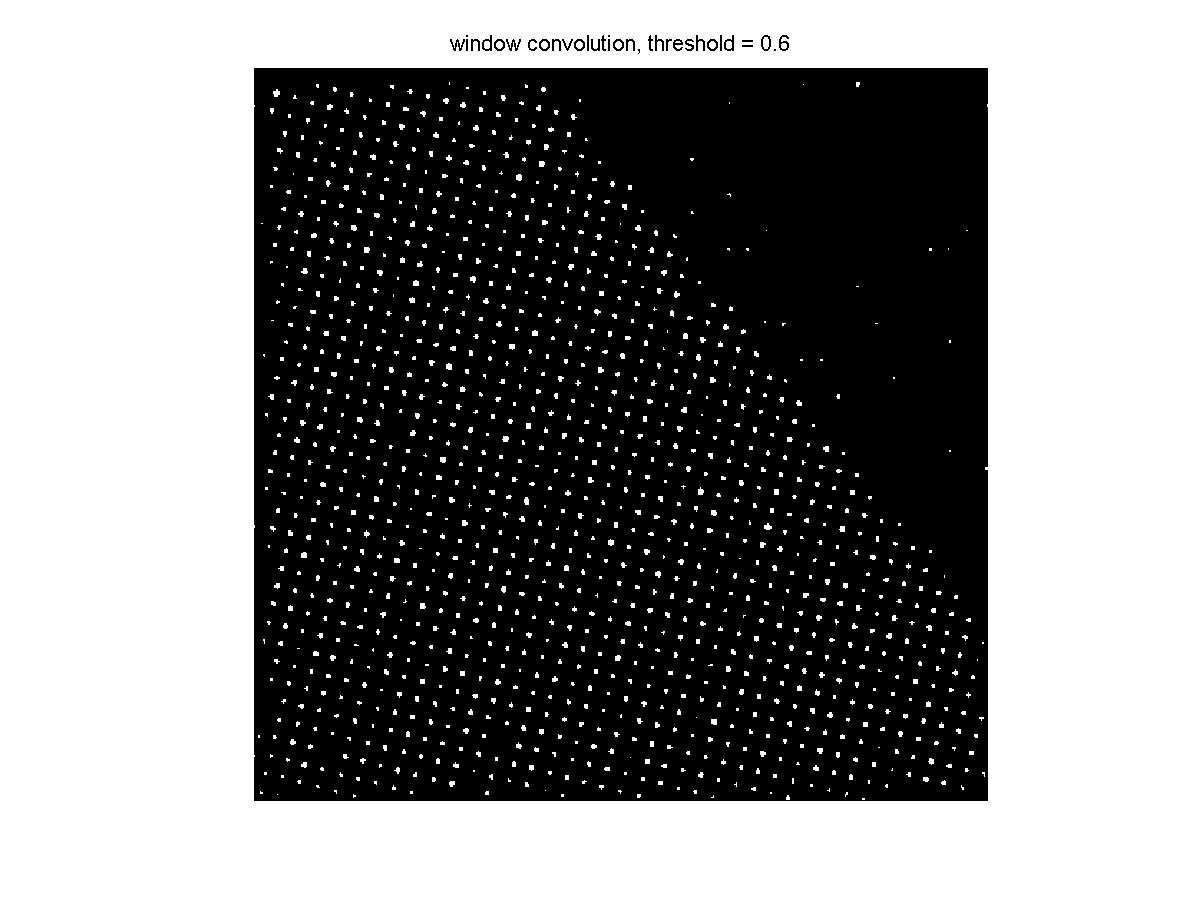** | **(c)**  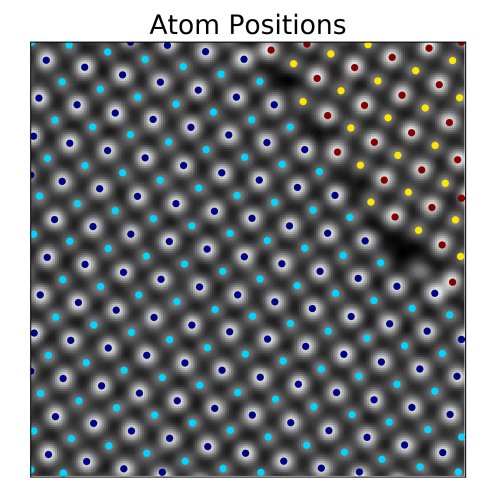 |
| --- | --- | --- |

**Figure S12. Atom-finding using existing techniques** – (a) Gaussian convolution, (b) window convolution, and (c) our proposed algorithm were applied to identify atoms in a simulated figure, shown in figure 3(i) without any noise. Gaussian convolution was unable to differentiate the two regions with different lattice structures and it identified all atoms under the same single class. The window convolution was able to identify only the atoms in one of the regions and all the atoms in the identified region were classified as the same kind of atom. In comparison, our algorithm is able to distinguish both the regions with different lattice structures in addition to the different kinds of atoms within each region.
